# Influence of Additive Manufacturing Parameters and Surface Treatments on Wettability of VPP Acrylic Resins

**DOI:** 10.3390/polym18141738

**Published:** 2026-07-15

**Authors:** María Jordá-Reolid, Ivan Dominguez-Candela, Mirko Kunowsky, Ignacio Sandoval-Pérez, Asunción Martínez-García

**Affiliations:** 1 AIJU Technological Centre, Avda. de la Industria, 03440 Ibi, Spain; mirkokunowsky@aiju.es (M.K.); nachosandoval@aiju.es (I.S.-P.); sunymartinez@aiju.es (A.M.-G.); 2 Technological Institute of Materials (ITM), Universitat Politècnica de València (UPV), Plaza Ferrándiz y Carbonell 1, 03801 Alcoy, Spain; ivdocan@doctor.upv.es

**Keywords:** additive manufacturing, vat photopolymerisation, hydrophobicity, contact angle, laser microtexturing, sandblasting

## Abstract

There is a growing industrial interest in the development of functional plastic surfaces with hydrophobic and easy-to-clean properties, particularly in manufacturing sectors where safety, hygiene, and durability are critical requirements. This work investigates the development of hydrophobic and superhydrophobic surfaces on acrylic resin components fabricated by vat photopolymerisation (VPP), using a high-performance Rigid 10K photopolymer. The influence of manufacturing parameters, namely layer thickness and build orientation, on initial wettability was first evaluated, showing that orientation plays a more relevant role than layer thickness in controlling the water contact angle. Subsequently, different surface modification strategies were explored, including femtosecond laser microtexturing, sandblasting, and physical vapour deposition (PVD) coatings. Preliminary results indicate that femtosecond laser texturing enables controlled modification of surface roughness and wettability, with strong dependence on laser fluence and pitch. Sandblasting significantly increases surface roughness, promoting hydrophobic behaviour through the generation of irregular topographies. In contrast, PVD coatings appear to modify wettability primarily through surface chemistry. Roughness analysis suggests that, although layer thickness governs the initial surface condition, post-processing treatments progressively dominate the final surface morphology. Ongoing work is focused on fully correlating roughness parameters, surface morphology, and wettability performance. Overall, the combination of VPP and tailored surface treatments presents a promising approach for functionalising polymeric surfaces for advanced engineering applications.

## 1. Introduction

Hydrophobicity and superhydrophobicity are key properties governing surface–liquid interactions in functional materials. The concept of superhydrophobicity is commonly associated with the Lotus effect, where hierarchical surface structures lead to extreme water repellence [1,2,3,4]. Recent studies have further emphasised the importance of durability and multifunctionality in practical applications [5].

Superhydrophobic surfaces are defined by contact angles exceeding 150°, low roll-off angles, and minimal contact angle hysteresis [6,7]. Acrylic resins used in vat photopolymerisation exhibit moderate surface energy, requiring structural or functional surface modification to achieve hydrophobic or superhydrophobic behaviour. Such modifications typically involve the introduction of micro- or nano-scale surface features inspired by natural superhydrophobic systems. Superhydrophobic behaviour in polymeric systems is typically achieved through a combination of surface chemistry modification and hierarchical structuring [8].

Due to their benefits, including self-cleaning, reduced corrosion and wear (in case of metals), anti-icing performance, improved fluid flow, and enhanced hygiene, hydrophobic and superhydrophobic surfaces remain an active area of research across multiple technological sectors.

Additive manufacturing (AM), defined by ISO/ASTM 52900:2021 [9], has emerged as a key enabling technology for producing complex geometries and customised components, facilitating the integration of functional surface properties [10,11]. The possibility of enhancing AM-produced components through post-processing surface modification has motivated extensive research in recent years. Among these methods, femtosecond laser modification is one of the most widely explored techniques for generating hydrophobic and superhydrophobic surfaces. By recreating hierarchical roughness similar to that of natural non-wetting surfaces, femtosecond laser modification enables improvements in water repellence and self-cleaning behaviour [12]. On the other hand, metallic coatings obtained by physical vapour deposition (PVD) represent a relevant strategy for tailoring surface properties. The PVD process is based on the physical vaporisation of a solid material, typically metals or alloys, using techniques such as sputtering or cathodic arc evaporation, followed by the condensation of the vapour onto a substrate under vacuum conditions. This approach enables the production of thin, dense coatings with high adhesion, while offering precise control over chemical composition and microstructure. Due to the relatively high energies of the arriving species, PVD-deposited metallic coatings exhibit refined microstructures and superior properties compared to those produced by conventional thermal or electrochemical techniques [13,14,15,16].

On the other hand, sandblasting is a high-intensity treatment that uses an abrasive material, α-aluminium oxide (corundum) (Al_2_O_3_). This process can create both very rough and finely polished surfaces, depending on the grain size of the corundum used. It is also used as a combined pretreatment with coatings on steels and metals to prevent corrosion [17,18], but it has been considered that the microetching or the wear it produces on polymeric surfaces [19] can also contribute to the hydrophobicity.

Within this framework, VPP technologies have emerged as highly promising approaches for the fabrication of complex, customised, and high-resolution polymer components. VPP technology is widely recognised for its high resolution and excellent surface finish compared to other additive manufacturing techniques [20]. Recent advances in photopolymer chemistry have enabled the development of engineering-grade resins, such as Rigid 10K, which exhibit enhanced stiffness, thermal resistance, and dimensional stability compared to other conventional photopolymers [21,22].

This is particularly relevant for sectors requiring low-to-medium production volumes, high geometrical complexity, and rapid design adaptation. The optimisation of process parameters, such as exposure time, layer thickness, and manufacturing orientation, plays a critical role in determining the final mechanical performance of VPP-fabricated parts [23,24].

Beyond mechanical performance, recent developments in additive manufacturing have increasingly focused on the integration of functional surface properties, such as hydrophobicity, directly into printed components. Hydrophobicity in VPP components can be achieved through material-driven approaches (e.g., incorporation of hydrophobic moieties) or structure-driven approaches based on micro- and nano-scale surface texturing [24,25,26,27]. Recent studies have demonstrated that the design of microstructures in VPP, such as pillar geometries, has a direct impact on contact angle values, highlighting the importance of geometrical design in wettability control [28,29]. Furthermore, the final geometry of these structures is strongly dependent on the capabilities and limitations of the manufacturing process, establishing a direct relationship between fabrication technology, surface design, and wettability performance [29,30]. However, despite these promising strategies, the implementation of hydrophobicity in high-performance VPP materials remains limited. In particular, there is scarce literature addressing the combination of high mechanical stiffness (as provided by Rigid 10K-like resins), dimensional accuracy, and durable hydrophobic behaviour under real operating conditions. Establishing quantitative relationships between fabrication parameters, surface topology, and wettability remains a critical challenge for the scalable design of functional surfaces [27,31].

Laser microtexturing represents another particularly attractive post-processing method to produce hydrophobicity, due to the high dimensional accuracy and surface quality of printed parts. In this context, laser processing parameters determine key design characteristics, such as texture depth, feature spacing, and surface roughness, which in turn govern the wetting response of the modified surface. Although studies have demonstrated that laser-textured polymer surfaces can achieve water contact angles exceeding 150°, particularly when combined with low-surface-energy surface chemistry or post-treatment [32,33,34], most of these studies have focused on conventional polymers (with limited attention given to photopolymers and parts produced via VPP) [35].

Therefore, the development of hydrophobic surfaces cannot be considered solely as a material selection problem, but also as a design and manufacturing challenge in which the final functionality depends on the interaction between fabrication technology, surface architecture, and post-processing strategy. Understanding these interactions is essential for the effective design of functional surfaces produced by additive manufacturing.

In this context, the present research focuses on the additive manufacturing of test specimens using vat photopolymerisation, followed by surface modification through different microstructuring and coating techniques, with the goal of achieving hydrophobic or superhydrophobic behaviour in acrylic resin parts. The study evaluates how these modifications influence surface energy and contact angles, comparing the effectiveness of various treatments.

## 2. Materials and Methods

### 2.1. Materials

The photopolymer resin used in this study was a commercial resin filled with glass fibre, Rigid 10K, supplied by FormLabs (Formlabs Inc., Somerville, MA, USA). According to the supplier, the mechanical properties are summarised in Table 1, showing a heat resistance material suitable for wide applications [21].

### 2.2. Printing of Samples

With the aim of studying different surface modifications to enhance hydrophobicity, rectangular samples (20 × 30 × 1 mm^3^) were designed using Solid Works 3, 2021 version (Dassault Systèmes, Vélizy-Villacoublay, France), and exported in stereolithography interface format (STL). Samples were fabricated using a 3BL vat photopolymerisation (VPP) printer (Formlabs Inc., Somerville, MA, USA) operating at a wavelength of 405 nm. Printing parameters are included in Table 2. Both layer thickness and build orientation with respect to the horizontal plane were investigated, and the samples were denoted according to layer thickness and build orientation, e.g., 50_A0 for the sample manufactured with a 50 μm layer thickness and 0° orientation. After VPP fabrication, the samples were removed from the build platform, subjected to a washing process for 3 min in isopropanol, followed by 20 min of ultrasonic cleaning. Subsequently, the samples were post-cured in an ultraviolet (UV) chamber for 60 min at 70 °C.

### 2.3. Surface Modification

#### 2.3.1. Femtosecond Laser Method

**Figure 1:**
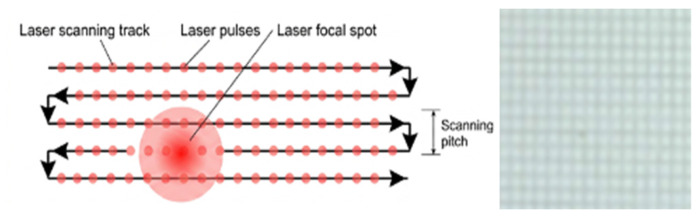
Scheme of femtosecond laser parameters. The arrows indicate the scaning direction, and the red dots the laser pulses.

Femtosecond laser texturing was conducted on the sample surfaces using a Georg Fischer AG laser system (Schaffhausen, Switzerland), with a maximum single-pulse energy of 150 μJ and a wavelength of 1064 nm. The laser power and repetition frequency were set at 7.5 W and 500 kHz, respectively, with a translation velocity of 2000 mm·s^−1^. An array of orthogonal micro-grooves arranged in a square scanning pattern (Figure 1) was generated with a pitch values of 0.03, 0.05, 0.07, and 0.1 mm at two different fluence values (pulse energy per square) of 0.056 μJ·cm^−2^ (referred to as F1) and 0.092 μJ·cm^−2^ (referred to as F2). This was done to assess the influence of laser parameters on surface topology and water contact angle. This treatment was carried out on three test specimens to ensure the repeatability of the tests on different specimens.

#### 2.3.2. Sandblasting Method

The rectangular samples were sandblasted with Al_2_O_3_ particles (particle size of 100 mm) at a pressure of 7 bar for 3 s, projected at a distance of 20 cm with an inclination of 90° applying 1 and 4 cycles of abrasion to study the effect on the surface. The average number of particles was found to be 100 μm. Afterwards, the samples were cleaned with compressed air in order to avoid any additional modification of the surface. This approach was selected as a more sustainable process than ultrasonic cleaning baths with ethanol or acetone, which have been reported elsewhere [36].

#### 2.3.3. Physical Vapour Deposition (PVD)

A TiN coating was deposited by reactive physical vapour deposition (PVD) using a Univex 400 system (Leybold, Cologne, Germany) equipped with electron-beam evaporation. Prior to deposition, the chamber was evacuated to a base pressure below 1 × 10^−6^ mbar. Subsequently, nitrogen was introduced at a flow rate of approximately 5 mL/min while maintaining the turbomolecular pump in operation, resulting in a working pressure of ~4 × 10^−4^ mbar. This controlled sub-atmospheric nitrogen atmosphere was employed to promote the formation of TiN during deposition and to compensate for possible dissociation of the evaporated species. Titanium nitride was evaporated using an electron-beam source operated at an accelerating voltage of 9.9 kV, with the evaporation rate controlled via the beam current (maximum intensity up to 4%). The substrates were mounted on a rotating holder (15 rpm) to ensure coating uniformity. The deposition rate was on the order of 0.3 Å/s, with total process times between 40 and 50 min. Under these conditions, TiN coatings with thicknesses in the range of approximately 0.39–0.44 µm were obtained, as determined by profilometry (KLA Tencor D-600, San Jose, CA, USA).

### 2.4. Surface Characterisation

#### 2.4.1. Contact Angle Measurements

The contact angles on the surfaces, to assess the hydrophobicity properties before and after the microtexturing, shot blasting, and after PVD coating treatments, were measured at 25 °C using Ramé-Hart 100-0 equipment (Succasunna, NJ, USA), placing 1 μL drops of double-distilled and deionised water on the surface. The values of the contact angles were measured at 30 s after the droplet was deposited. The measurement of five drops was performed for each sample, taking fifteen measurements in each drop, and the contact angle was calculated as the average of these measurements.

#### 2.4.2. Attenuated Total Reflection–Fourier Transform Infrared Spectroscopy (ATR-FTIR)

To determine the chemical nature of the resin and the PVD coating, attenuated total reflection–Fourier transform infrared (ATR-FTIR) spectroscopy was carried out. Spectra were recorded using a FT/IR-4X of Jasco (Tokyo, Japan). Data were collected as the average of 16 scans between 4000 and 500 cm^−1^ with a spectral resolution of 4 cm^−1^ and the measurement was performed at room temperature.

#### 2.4.3. Scanning Electron Microscopy (SEM)

The surfaces of the specimens before and after the different treatments were observed by scanning electron microscopy (SEM), using a JEOL JSM-840 SEM system (Tokyo, Japan) with an electron beam acceleration voltage of 20 kV. Prior to SEM analysis, the samples were coated with a thin layer of Ag using a current of 8 mA over 120 s under vacuum in a sputter-coated EM MED020 from Leica Mycrosystems (Wetzlar, Germany).

#### 2.4.4. Roughness Parameters

The roughness parameters R_a_ and R_z_ of the untreated samples and those treated with femtosecond laser texturing and corundum sandblasting were also determined. Surface roughness measurements were performed using a MarSurf PS10 profilometer (Mahr GmbH, Göttingen, Germany). The device is equipped with a stylus probe featuring a tip radius of 2 μm, which traverses the sample surface to capture its topographical features. A total evaluation length of 4 mm was used for each measurement, allowing the characterisation of surface irregularities and textures. The parameter R_a_ is the arithmetic mean roughness, which represents the average deviations of the surface profile from the mean line over the sampling length (peaks and valleys). R_z_ is the average roughness depth, calculated as the average distance between the five highest peaks and the five deepest valleys within the evaluation length. Therefore, it would be the average peak-to-valley height of the surface profile. The 3D maps were obtained with the KLA Tencor D-600 profilometer (San Jose, CA, USA), by scanning an area of 50 μm × 50 μm, with a scan line spacing of 1 μm, a scan speed of 1 μm/s, and a stylus force of 1 mg.

#### 2.4.5. Statistical Analysis

The influence of surface processing and post-processing treatments on the water contact angle (WCA) of the acrylic resin was evaluated using a series of general linear model (GLM)-based analysis of variance (ANOVA) approaches, adapted to the hierarchical structure of the experimental design.

The effect of sandblasting was analysed using a GLM-based ANOVA to quantify the influence of surface treatment conditions (no treatment, 1 pass, and 4 passes) on wettability. Manufacturing parameters, including layer thickness (50 and 100 μm) and build orientation (0°, 45°, and 90°), were included in the model as additional factors to account for their contribution to the initial surface state. Due to the hierarchical nature of the experimental design and to ensure statistical robustness, interaction terms were not included in the final model. The model used was as follows: WCA∼C(Treatment) + C(Layer Thickness) + C(Orientation).

The influence of femtosecond laser texturing on surface wettability was analysed using a factorial ANOVA framework. Laser processing parameters, specifically fluence (F1 and F2) and microtexture pitch (0.03–0.10 mm), were considered the primary factors governing surface modification. Layer thickness and build orientation were included as blocking factors to account for the underlying manufacturing conditions. A full factorial interaction between fluence and pitch was included to capture coupled effects between laser energy input and surface geometry. Model assumptions of normality and homoscedasticity were verified prior to analysis. The final model used was as follows: WCA∼C(Fluence) × C(Pitch) + C(Layer Thickness) + C(Orientation).

All laser-textured specimens were subsequently coated with a TiN layer using physical vapour deposition (PVD). Since the coating was applied uniformly to all samples, it was not treated as an independent statistical factor. Instead, its effect is discussed qualitatively as a global surface modification contributing to changes in surface chemistry and wettability.

All statistical analyses were performed using a general linear model (GLM) framework. The significance level was set at α = 0.05 for all tests.

## 3. Results

### 3.1. Contact Angle Measurement

#### 3.1.1. Effect of Processing Parameters

The static water contact angle (WCA) was measured of samples manufactured using two layer thicknesses, 50 μm and 100 μm, and three build orientations, 0, 45, and 90°, as shown in Table 3. In all cases, WCA values below 52° were obtained, indicating the hydrophilic character of the printed acrylic resin. For samples manufactured with a layer thickness of 50 μm, the WCA decreased from 51.7° at a 0° build orientation to 27.8° at 90°, indicating a clear improvement in surface wettability as the build orientation increased. A comparable reduction was also observed for samples manufactured with a layer thickness of 100 μm, for which the WCA decreased from 42.8° at 0° to 24.6° at 90°. However, in this case, the sample printed at 45° showed a slightly higher WCA value, 47.4°, than that obtained at 0°, suggesting that the effect of build orientation may not be strictly linear for all layer thicknesses. Overall, the variation in WCA was similar for both layer thicknesses, with reductions of approximately 43–47% between 0° and 90°. The WCA values were generally slightly higher for the 50 μm layer thickness samples, except at 45°, although the differences between layer thicknesses were less pronounced than those observed between build orientations. These results suggest that build orientation has a stronger influence on surface wettability than layer thickness. It should also be noted that build orientation plays a key role in manufacturing 3D-printed parts. Several authors have reported that a 45° orientation can improve overall dimensional accuracy while providing a balanced mechanical response [37,38]. In addition, complex parts often require increased orientation along the *z*-axis and the use of supporting structures to enable resin drainage and achieve high-quality geometries. Based on these considerations and taking into account the influence of the processing parameters on WCA, further characterisation will be focused on printed parts at a 45° build orientation with layer thicknesses of 50 μm and 100 μm, as these conditions are representative of a wide range of printed geometries.

#### 3.1.2. Influence of Femtosecond Laser Texturing

**Figure 2:**
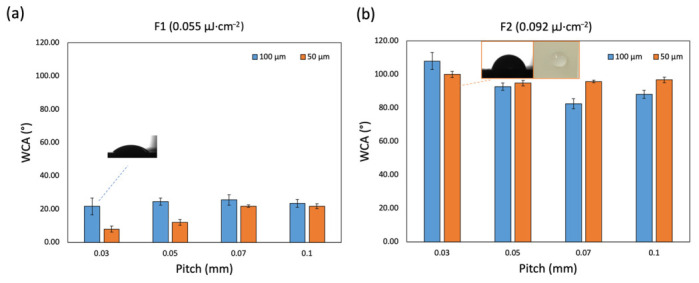
Water contact angle values of samples manufactured with a 45° build orientation and treated by femtosecond laser texturing as a function of layer thickness and pitch, using two laser fluence values: (**a**) 0.055 J·cm^−2^ and (**b**) 0.092 J·cm^−2^. Data are expressed as mean ± standard deviation.

The influence of the microtexturing pattern and processing parameters was evaluated on the wettability of the surfaces of samples printed at a 45° build orientation. The effect of the two laser fluences, F1 and F2, and pitch, applied by femtosecond laser texturing on samples manufactured with different layer thicknesses and build orientations, is shown in Figure 2. Regarding laser fluence, the higher the applied laser fluence, the more hydrophobic the surface behaviour observed across the whole pitch range studied. In this regard, two effects were identified depending on the laser fluence applied.

On one hand, the sample treated with lower laser fluence, F1 (0.055 μJ·cm^−2^), exhibited a more hydrophilic surface compared to the unmodified samples (see Table 3). The WCA decreased from 32.8° to 8–21.7° for samples manufactured with a 50 μm layer thickness, and from 47.4° to 21–25° for those manufactured with a 100 μm layer thickness. These results suggest that the amount of irradiated energy is not sufficient to cause surface modifications capable of increasing hydrophobicity, with the opposite behaviour being observed. From a wetting theory perspective, the behaviour observed at F1 is qualitatively consistent with the Wenzel model. Since the untreated composite exhibited hydrophilic behaviour (WCA < 90°), the marked reduction in WCA after laser texturing suggests that well-defined laser-generated microstructures facilitate liquid penetration into the surface texture, increasing the effective solid–liquid contact area. Therefore, the wetting behaviour observed at F1 is more consistent with a Wenzel-like regime than with a Cassie–Baxter state [39].

By contrast, irradiation at the higher fluence, F2 (0.092 μJ·cm^−2^), produced the opposite effect on the surface behaviour. In this case, the treated samples showed increased hydrophobicity compared with the untreated ones, with WCA values of at least 82° in all cases and reaching values of approximately 100–108°. In particular, an increase of 135–188% and 192–205% was observed for the 50 μm and 100 μm layer thickness samples, respectively. In contrast to the lower fluence treatment, the 50 μm layer thickness did not significantly affect the WCA, whereas a greater influence was observed for the 100 μm samples, demonstrating that the manufacturing parameters also affect the final surface wettability. It is noteworthy that no clear correlation was observed between surface roughness and wettability under the F2 treatment. The 0.03 mm pitch condition exhibited the highest WCA values despite presenting the lowest roughness values (R_a_ = 1.580 μm; R_z_ = 10.380 μm) compared to 0.05 mm pitch (R_a_ = 4.957 μm; R_z_ = 25.731 μm), as shown in Table 8 (Section 3.4.1). Nevertheless, increasing the pitch from 0.05 to 0.1 mm resulted in progressively higher roughness values, while the WCA remained nearly constant or showed a slight decrease. These results indicate that the wettability response cannot be explained only by the roughness parameters measured in this study. According to classical wetting theories, surface roughness can modify the apparent contact angle through Wenzel or Cassie–Baxter mechanisms [40]. However, the absence of a positive correlation between roughness and WCA suggests that topographical effects alone do not govern the observed wetting behaviour. Furthermore, the higher contact angles obtained for the smallest 0.03 mm pitch condition indicate that additional surface characteristics may contribute significantly to the wettability response.

Therefore, the observed behaviour is likely the result of a combination of factors, including the specific morphology generated by laser irradiation and possible changes in surface physicochemical properties [41]. Nevertheless, the available experimental data do not allow the dominant wetting regime to be unequivocally established. Additional analyses, such as contact angle hysteresis measurements and surface chemical characterisation, would be required to clarify the mechanisms responsible for the observed hydrophobicity [42]. It should be noted that other authors have also reported two regimes influenced by laser irradiation parameters. Pazokian et al. [43] found an initial decrease in WCA up to a specific laser fluence, followed by an increase once this threshold was exceeded, on the polyethersulfone (PES) surface. Therefore, these results demonstrate the possibility of tuning the surface wettability from hydrophilic to hydrophobic by adjusting both the applied laser fluence and pitch.

The statistical analysis (Table 4) revealed that laser fluence is the dominant parameter governing the wettability response of the laser-textured surfaces. Fluence exhibited a highly significant effect on the water contact angle (F = 88.63, *p* < 0.001), indicating a strong energy-driven transition in surface wettability. In contrast, microtexture pitch did not show a statistically significant effect (*p* = 0.93), suggesting that geometric spacing alone does not govern the global wetting behaviour within the investigated range. Although the fluence–pitch interaction was not statistically significant (*p* = 0.498), local variations in contact angle were observed depending on specific combinations of parameters, indicating a non-uniform response across the experimental domain. Manufacturing parameters, such as layer thickness, also exhibited statistically significant effects (*p* < 0.05), although its influence was considerably lower than that of laser fluence. This suggests that the initial surface state still contributes to the final wettability response but does not dominate the laser-induced behaviour.

#### 3.1.3. Effect of Sandblasting

**Figure 3:**
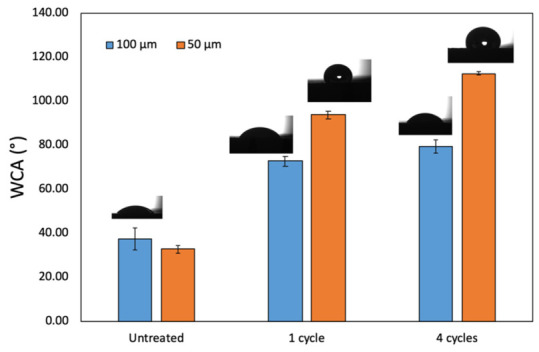
Water contact angle values of samples manufactured with a 45° build orientation as a function of layer thickness and sandblasting treatment. Data are expressed as mean ± standard deviation.

The wettability behaviour of sandblasted surfaces is shown in Figure 3. As can be observed for both layer thicknesses, the untreated samples exhibited low WCA values (32–37°) characteristic of printed wettable surfaces, as aforementioned. After sandblasting, the WCA increased with the number of cycles applied, with a more pronounced increase after one cycle and a lesser increase after four cycles. This trend is in line with other studies reported in the literature, where sandblasting has been shown to produce hydrophobic surfaces due to changes in surface roughness. Nevertheless, this effect is strongly linked to the mechanical properties of the materials studied, such as yield strength, and could lead to different behaviours depending on the polymer under study [44]. As observed for Rigid 10K, all values exceeded 70° regardless of layer thickness, reaching the hydrophobicity threshold (>90°) with values of 93–112° after one and four sandblasting cycles for samples printed with a 50 μm layer thickness. In this regard, it is noted that layer thickness affected the WCA behaviour, with higher values observed for samples printed at 50 μm than for those printed at 100 μm. Comparing the wettability results with roughness data (further explained in detail in Section 3.4.2, Table 6), the variations in R_a_ and R_z_ values with sandblasting cycles as well as layer thickness are considered similar. For example, layer thickness of 50 μm presented changes in Ra values from 6.37 μm (one cycle) to 5.75 μm (four cycles) and for 100 μm changes from 5.98 μm to 5.71 μm, with no changes significant according to standard deviation, with a similar trend for Rz values. This lack of direct correlation between R_a_ and R_z_ and wettability indicates that roughness, as described by these average amplitude parameters, cannot account alone for the marked increase in hydrophobicity observed after sandblasting. This is consistent with previous studies showing that wetting behaviour often cannot be predicted from simple roughness parameters alone [45]. The increase in water contact angle with the number of sandblasting cycles for both layer thicknesses could be interpreted as arising from a combined effect of roughening and a redistribution of roughness scales and microrelief shapes that are not fully reflected by R_a_ and R_z_, rather than from geometrical factors quantified by these average parameters alone [46]. Therefore, this treatment represents an excellent alternative for achieving hydrophobic surface at relatively low processing cost and could be applied in industrial processes to induce self-cleaning properties.

The statistical analysis (Table 5) confirmed that sandblasting treatment is a highly significant factor affecting surface wettability (F = 12.78, *p* = 0.0011), indicating that the number of cycles plays a dominant role in modifying the water contact angle. However, the experimental results show that the effect of increasing the number of cycles is not strictly linear. Although the treatment significantly modifies wettability overall, the transition from one to four cycles does not always result in a proportional increase in contact angle. This suggests that the effect of sandblasting depends on the initial surface state defined by layer thickness and build orientation. In particular, layer thickness also showed a statistically significant influence (*p* = 0.0186), indicating that the baseline surface morphology affects the efficiency of the sandblasting process. These results indicate that sandblasting modifies wettability through a non-linear and geometry-dependent mechanism, where the number of cycles determines the overall magnitude of the effect, but its local response is modulated by the initial surface structure.

#### 3.1.4. Effect of PVD

**Figure 4:**
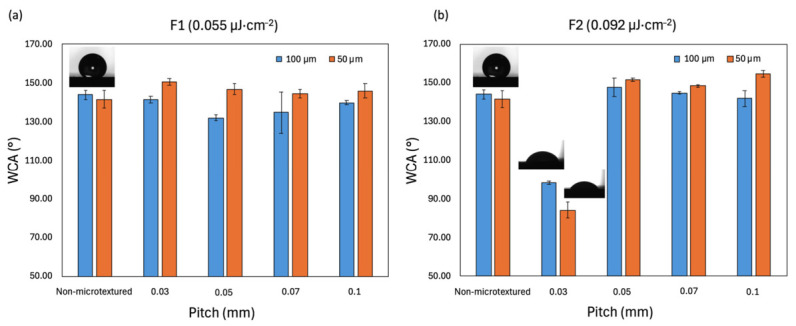
Water contact angle values of PVD-coated surfaces as a function of laser microtexturing pitch and layer thickness for two laser fluence values: (**a**) 0.055 J·cm^−2^ and (**b**) 0.092 J·cm^−2^. Data are expressed as mean ± standard deviation.

The influence of the PVD coating on the wettability of the laser-textured surfaces as a function of laser fluence, pitch, and layer thickness is shown in Figure 4. As can be observed, the WCA is affected by all three parameters, following a non-linear trend. The non-microtextured reference samples exhibited WCA values between 142° and 144°, approaching the threshold commonly associated with superhydrophobic surfaces [47]. These values are significantly higher than the static contact angle reported for TiN-coated glass (73.5°) and even exceed those reported for modified F-TNTs/TiN coatings developed for superhydrophobic applications [41]. Compared with the corresponding non-coated samples, the PVD-coated surfaces exhibited a substantial increase in WCA under all processing conditions, highlighting the strong influence of the coating on the final wetting behaviour. While some texturing conditions resulted in lower WCA values than the non-microtextured reference, pitches of 0.05 mm and above generally maintained or slightly increased the hydrophobicity, reaching values between 150° and 154°. These observations indicate that the final wetting behaviour is governed by both the surface chemistry introduced by the TiN coating and the morphology of the laser-generated microstructures. In this regard, FTIR analysis of the TiN-coated samples was performed to investigate the chemical modifications associated with the PVD treatment. The results, discussed in Section 3.2, provide further evidence that the coating alters surface chemistry.

The influence of pitch was dependent on laser fluence. At the lower fluence, F1 (0.055 μJ·cm^−2^), only moderate variations in WCA were observed throughout the investigated pitch range, suggesting that the wettability was primarily controlled by the TiN coating, whereas the laser-generated microstructures produced only minor pitch-dependent variations in the contact angle. In contrast, the effect of pitch became more evident at the higher fluence, F2 (0.092 μJ·cm^−2^). Interestingly, the lowest WCA values were obtained for the surfaces textured with a pitch of 0.03 mm, reaching approximately 84° and 98° for the 50 μm and 100 μm layer thicknesses, respectively. These values were considerably lower than those measured for pitches of 0.05 mm and above, where WCAs between 141° and 154° were obtained. The wettability trend was analysed together with the corresponding roughness data presented in Table 7, which will be further commented on in detail in Section 3.4.3. For example, the 50 μm layer thickness samples exhibited a reduction in roughness, R_a_ and R_z_, from 4.52 μm and 23.82 μm at a pitch of 0.05 mm, respectively, to 1.965 μm and 11.855 μm at a pitch of 0.03 mm. However, roughness measurements performed before and after PVD deposition showed only minor variations in the topographical parameters, as expected for this type of coating [48]. Similar observations have been reported for textured surfaces, where wettability was found to depend not only on roughness magnitude but also on the interaction between surface chemistry and microstructure geometry [49]. In the present study, the limited changes in roughness following PVD deposition, together with the marked differences in WCA between pitches, suggest that the TiN coating modifies the surface chemistry while the laser-generated texture governs how this modification is expressed in the final wetting response.

SEM observations described in Section 3.3 revealed that the combination of high fluence and small pitch (0.03 mm) promoted partial coalescence of adjacent laser tracks, resulting in a less distinct surface morphology. Such modifications of the surface architecture after PVD deposition may reduce the ability of the texture to maintain the favourable wetting state achieved for larger pitches. In contrast, pitches of 0.05 mm and above preserved a more clearly defined microstructure, with well-separated grooves and ridges that maintained the integrity of the laser-generated pattern. This more regular surface architecture is likely to promote a lower effective solid–liquid contact area and a more stable hydrophobic wetting state, resulting in substantially higher WCAs between 150° and 154°.

Overall, the results indicate that the TiN coating provides the primary contribution to the hydrophobic response through its surface chemistry, while the laser-generated microstructures modulate the final wettability depending on the pitch and fluence conditions. Under the most favourable conditions, F2 combined with pitches ≥ 0.05 mm enabled the formation of superhydrophobic surfaces (WCA > 150°), outperforming the corresponding non-coated conditions and demonstrating significant potential for self-cleaning applications [43].

The statistical analysis (Table 6) revealed that laser fluence is the most influential parameter affecting surface wettability (F = 198.4, *p* < 0.0001), indicating a strong energy-driven transition between hydrophilic and hydrophobic regimes after laser texturing and PVD coating. Microtexture pitch also showed a statistically significant effect (*p* < 0.0001), although its influence was strongly dependent on laser fluence, as indicated by a significant fluence × pitch interaction (*p* < 0.0001). This confirms that pitch does not act as an independent controlling parameter but rather modulates the surface response within a fluence-dependent regime. Layer thickness exhibited a statistically significant effect (*p* = 0.0002), demonstrating that the initial manufactured morphology influences the final wettability even after laser processing and coating. The significant fluence × pitch interaction indicates that the effect of geometric structuring is highly dependent on laser energy input, supporting the existence of distinct wetting regimes rather than a continuous linear response.

### 3.2. Attenuated Total Reflection–Fourier Transform Infrared Spectroscopy (ATR-FTIR) Mesurements

**Figure 5:**
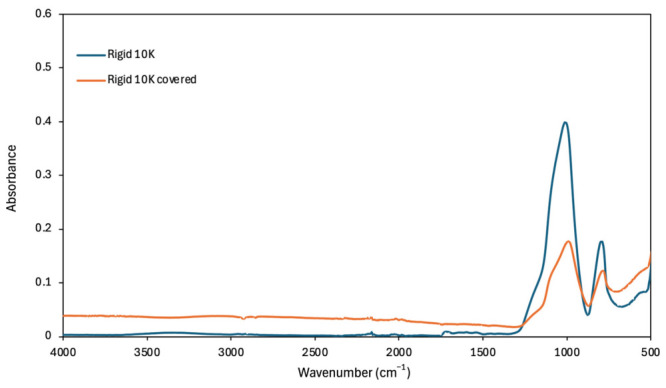
Rigid 10K FTIR spectrum and Rigid 10K PVD-coated FTIR spectrum.

Figure 5 shows a comparative graph of the infrared spectra of the resin used (Rigid 10K) in the manufacture of the specimens used for the present study, compared with the specimens coated with PVD.

The FTIR spectrum of the Rigid 10K resin shows the characteristic bands of an acrylic polymer network reinforced with an inorganic filler. A peak is observed at approximately 2956 cm^−1^, corresponding to the aliphatic C–H stretching vibrations of the methacrylate polymer chains, along with a strong band at ~1714 cm^−1^ assigned to the stretching of the carbonyl (C=O) group, typical of ester functionalities present in the photopolymerised matrix. Additionally, a signal at ~1639 cm^−1^ is observed, associated with residual vibrations of acrylate C=C bonds, indicating a low contribution after polymerisation. The region around ~1165 cm^−1^ corresponds to C–O–C stretching vibrations characteristic of ester linkages in the polymer network, while the band at ~1045 cm^−1^ is assigned to Si–O–Si vibrations, confirming the presence of the glass fibre filler. In comparison with bibliographic data [50] for this resin, the band positions are in close agreement, particularly in the carbonyl (~1720 cm^−1^), C–H (~2957 cm^−1^), C–O–C (1150–1250 cm^−1^), and Si–O–Si (1100–1000 cm^−1^) regions, confirming the same chemical structure of the Rigid 10K resin. On the other hand, the PVD TiN surface coating does not introduce new chemical bands; however, it causes variations in relative intensity and slight baseline distortions in several spectral regions. These effects are attributed to optical reflection/absorption of the metallic coating, with no evidence of chemical modifications in the functional groups of the underlying resin. This may be due to the very low thickness of the coating, which is not detected by IR radiation. A significant contribution from the Rigid 10K substrate is expected in the recorded spectrum, given that the TiN coating thickness (~0.44 µm) is comparable to or less than the standard ATR-FTIR penetration depth (0.5–3 µm). Accordingly, the absence of poorly resolved Ti–N bands simply reflects the limited surface sensitivity of the technique under these thickness conditions [51].

### 3.3. Surface Morphology

#### 3.3.1. Effect of Femtosecond Laser Texturing on Surface Morphology

**Figure 6:**
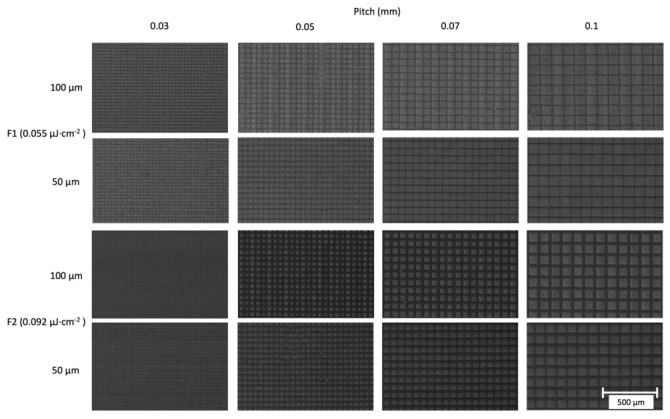
SEM images of the printing and laser parameters’ effect on the surface morphology of Rigid 10K at 100× and 5 kV: printing layer thickness (50 and 100 μm), laser fluence energy (0.055 μJ·cm^−2^ and 0.092 μJ·cm^−2^), and pitch (0.03, 0.05, 0.07, and 0.1 mm).

The influence of the applied pattern and laser parameters on the surface morphology of the VPP-printed samples is shown in Figure 6. As expected, the non-microtextured sample exhibits a flat surface with no visible pattern for both printing layer thicknesses studied, as confirmed in Figure S1. After femtosecond laser processing, a well-defined array of orthogonal micro-grooves was observed in all samples, except for specific parameters described below. In general, the surface showed a regular lattice attributed to the selected pitch values, similar to those reported in polymers and metals [52]. The groove widths of the microtextures were measured by profilometry, confirming the nominal pitch values of 0.03, 0.05, 0.07, and 0.1 mm. As can be observed from the SEM images, increasing the laser fluence from 0.055 μJ·cm^−2^ to 0.092 μJ·cm^−2^ lead to wider and more clearly defined grooves. Comparing this morphology with the WCA values suggests that the lower laser fluence, F1 (0.055 μJ·cm^−2^), could facilitate droplet spreading compared with the non-microtextured sample, suggesting that the liquid penetrates into the micro-cavities, thereby enhancing wettability. Conversely, the higher pulse energy, F2 (0.092 μJ·cm^−2^), produced more pronounced grids with wider grooves, which seems to modify the effective contact between the surface and droplet [53], thus allowing a transition from a hydrophilic state in the non-treated samples to a hydrophobic state. It is worth noting that surfaces with a 0.03 mm pitch for samples manufactured with both layer thicknesses appear less sharply defined than those produced with larger pitches, according to the SEM images. This surface morphology could be attributed to overlap between neighbouring scan tracks, which could cause morphological changes, affecting the intended pattern [54]. This observation is consistent with the roughness trend discussed in Section 3.4.1 and summarised in Table 8. For example, the roughness parameters for the 50 μm layer thickness decreased in R_a_ and R_z_ values from 5.52 μm and 25.5 μm at a 0.05 mm pitch to 1.580 μm and 10.380 μm at a 0.03 mm pitch, respectively. The combination of 0.092 μJ·cm^−2^ and 0.03 mm pitch for both layer thicknesses likely promotes partial coalescence of adjacent grooves, reducing the contrast of the original pattern. Despite this, the resulting textured surface still provides micro-scale features to maintain hydrophobic contact behaviour between the droplet and the surface (100–108°).

#### 3.3.2. Effect of Sandblasting on Surface Morphology

**Figure 7:**
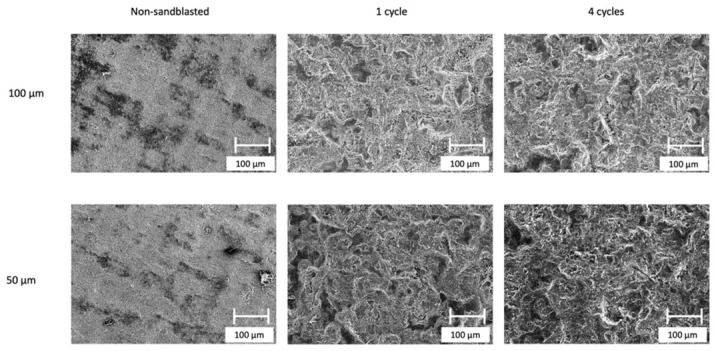
SEM images of sandblasted printed parts as a function of printing layer thickness (50 and 100 μm) and number of sandblasting cycles.

The SEM observations of sandblasted surfaces at different printing layer thicknesses and after different numbers of sandblasting cycles are shown in Figure 7. The non-sandblasted surfaces exhibited a relatively homogenous surface for both layer thicknesses, typical of printed VPP materials, with no pronounced irregularities [55]. Only slight differences in surface morphology were observed between both layer thicknesses, with the 100 μm samples exhibiting somewhat more pronounced irregularities. This is consistent with the literature, where lower printing resolution has been associated with more evident layer-related defects compared with thinner layers [56]. The application of sandblasting cycles resulted in highly rough surfaces with depressions and ridges for all samples, which can be ascribed to material removal and deformation due to the impact of abrasive particles. This morphology suggests that, despite the initial previous more homogenous surface observed for the 50 μm samples, sandblasting could modify the surface to some extent, creating more irregularities in both one and four cycles, with no clear differences between them. In contrast, the 100 μm samples showed less pronounced differences between the pre- and post-treatment surfaces. This morphology supports the increase of hydrophobicity observed for the 50 μm layer thickness samples. It should be pointed out that no evident surface contamination was found after the blasting treatments, even though the possibility that a negligible amount of abrasive particles remained attached to the surface cannot be ruled out. Nevertheless, our results are in agreement with those reported for other polymeric materials, such as acrylonitrile butadiene styrene (ABS), poly(methyl methacrylate) (PMMA), polypropylene (PP), and polycarbonate (PC), which were also treated by sandblasting under different parameters using alumina particles [44,57]. In these studies, no appreciable effect of residual particles on the wettability properties was reported.

**Figure 8:**
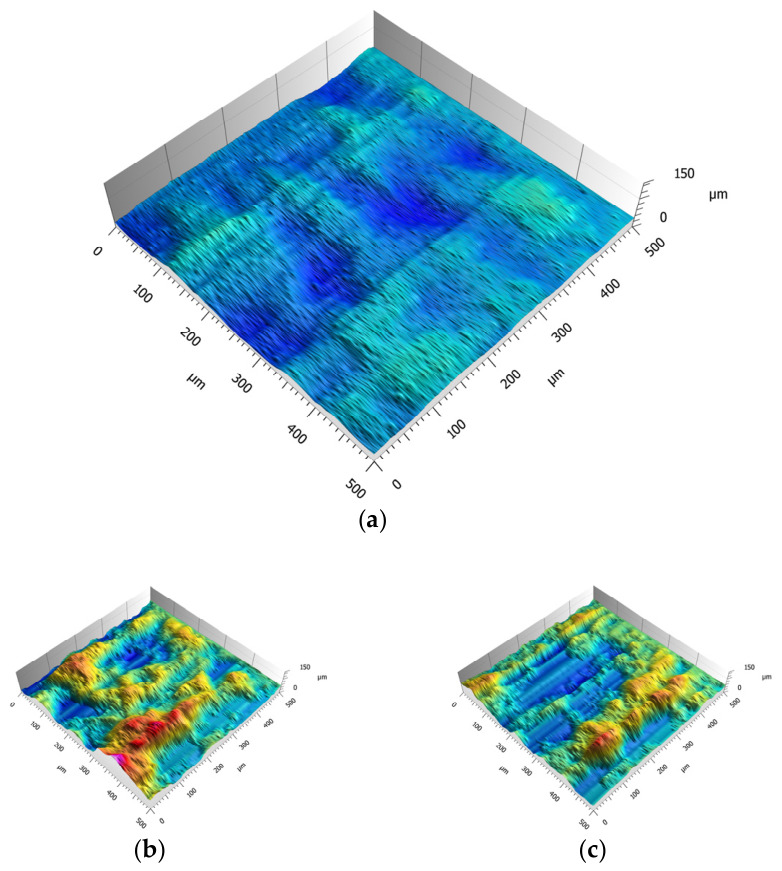
3D maps of the samples with a 50 μm layer thickness: without sandblasting (**a**), with one sandblasting cycle (**b**), and with four sandblasting cycles (**c**).

To better understand the wettability evolution induced by sandblasting, 3D maps were obtained for the samples with a 50 μm layer thickness, as this condition exhibited the highest WCA values while following the same increasing trend with the number of sandblasting cycles observed for the 100 μm samples. As shown in Figure 8, the untreated surface displayed a relatively smooth and continuous topography, which is consistent with its hydrophilic character and low WCA (40°). After one sandblasting cycle, the surface underwent a marked topographical modification, developing an irregular microtexture with pronounced peaks and valleys. This morphological change was accompanied by a substantial increase in WCA (~100°), suggesting that the generated surface features reduced the extent of liquid spreading. In addition, by increasing the number of sandblasting cycles to four, the WCA further enhanced to approximately 120°, although the R_a_ and R_z_ values remained comparable to those obtained for one cycle. As per the results, it is suggested that the more homogeneous and spatially organised microrelief observed for four cycles may have favoured partial air retention beneath the water droplet, thereby decreasing the effective solid–liquid contact area, which could be compatible with a Cassie–Baxter-like or mixed Cassie–Wenzel wetting state.

### 3.4. Roughness Parameters Mesurements

The surface roughness values are presented in Table 7, characterised by the parameters R_a_ and R_z_ for samples manufactured with two different layer thicknesses: 50 μm and 100 μm.

For the 50 μm layer thickness, a R_a_ value of 1.189 ± 0.053 μm is obtained, and an R_z_ value of 7.623 ± 1.151 μm is obtained. By increasing the layer thickness to 100 μm, a significant increase in roughness is observed. In this case, the R_a_ value amounts to 5.395 ± 0.596 μm, which increases more than four times compared to the sample made with 50 μm layers. Accordingly, the R_z_ parameter is 22.252 ± 1.760 μm, evidencing a greater separation between the maximum peaks and the minimum valleys of the surface profile.

From these data, it is concluded that increasing the layer thickness leads to greater surface roughness, as reflected by both R_a_ and R_z_ values. However, the corresponding increase in WCA from 32.8° (50 μm) to 47.4° (100 μm) does not follow the trend expected from roughness effects alone. According to classical wetting theories, surface roughness can amplify the intrinsic wetting behaviour of a material, leading to enhanced hydrophilicity for surfaces exhibiting contact angles below 90° [41]. Therefore, the increase in WCA observed for the 100 μm specimens suggests that the wetting response is not explained only by the magnitude of the roughness parameters. This behaviour may be associated with differences in the surface morphology generated during the VPP. In VPP, the surface texture is affected by process-related factors, such as the layer-wise curing strategy, layer thickness, build orientation, cure depth, and staircase-related effects [58]. Therefore, the increase in contact angle observed for the 100 μm specimens is likely related to changes in the surface texture associated with the larger layer thickness, which may partially counteract the hydrophilicity enhancement expected from the roughness effect. These findings indicate that the relationship between roughness and wettability is not straightforward and that R_a_ and R_z_ alone are insufficient to fully describe the surface characteristics controlling the water contact angle.

#### 3.4.1. Effect of Femtosecond Laser on Roughness Parameters

Table 8 presents the surface roughness values for samples manufactured with two different layer thicknesses, 50 μm and 100 μm, and treated with the femtosecond laser using four different pitch values with the F2 fluence (0.092 μJ·cm^−2^) texturing, selecting the latter because it yielded the lowest wettability for the intended application.

For both layer thicknesses, 50 μm and 100 μm, femtosecond laser texturing with a pitch of 0.03 mm resulted in the lowest roughness values, with a R_a_ values between 1.580 μm and 1.591 μm and R_z_ values between 9.963 μm and 10.380 μm, respectively. These values are slightly higher than those previously obtained in specimens manufactured at 50 μm without femtosecond laser texturing, indicating that a reduced pitch generates a fine and relatively homogeneous texture without introducing high-amplitude irregularities. However, when the pitch was increased to 0.05 mm, a considerable increase in roughness was observed. For this configuration, the R_a_ values were between 4.957 μm and 5.520 μm, while R_z_ reached values close to 25 μm, reflecting the appearance of much more marked surface profiles. This increase suggests that, from this spacing onwards, femtosecond laser texturing introduces more clearly defined discontinuities on the surface, according to SEM images where well-defined laser-generated microstructures were observed. For pitches of 0.07 mm and 0.10 mm, the roughness remained high, with some variations. In general, R_a_ increased slightly with pitch, reaching its maximum values at 0.10 mm, while R_z_ remained within a high range. This indicates that the extreme irregularities introduced by femtosecond laser texturing are maintained and, in some cases, intensified.

A relevant aspect is that, after femtosecond laser texturing, the differences between the 50 μm and 100 μm layer thicknesses were significantly reduced, especially for larger pitches. Unlike what was observed in untreated surfaces, where the effect of layer thickness was dominant, in this case the final topography is mainly governed by femtosecond laser texturing, which partially masked the influence of the initial roughness associated with the manufacturing process. However, for the smallest pitch (0.03 mm), the surfaces maintained relatively low and very similar R_a_ and R_z_ values for both layer thicknesses, indicating that this femtosecond laser texturing condition produces a controlled surface texture with lower dependence on the initial roughness. The SEM images suggest that, under F2 (0.092 μJ·cm^−2^), the reduced pitch favours partial overlap between adjacent laser-induced features, leading to a less pronounced periodic pattern reflected by its lower roughness values. Thus, the higher WCA obtained at 0.03 mm cannot be fully explained by the measured R_a_ and R_z_ values. Rather, this result points to the contribution of surface features beyond conventional roughness amplitude parameters, including the specific femtosecond-laser-induced morphology and possible changes in surface physiochemistry properties [42]. These effects may alter the surface energy and, consequently, the wetting response. However, based on static contact angle and roughness measurements, the dominant mechanism cannot be unambiguously determined. Further contact angle hysteresis measurements and surface chemical analyses would be required to decouple the contribution of topographical and physicochemical effects.

#### 3.4.2. Effect of Sandblasting on Roughness Parameters

In parallel to femtosecond laser texturing, a sandblasting treatment was applied to specimens manufactured with a layer thickness of 50 μm and 100 μm, evaluating the effect of one and four sandblasting cycles on surface roughness. The values obtained for R_a_ and R_z_ are collected in Table 9 and compared with the results previously obtained for untreated specimens.

The specimens manufactured with a layer thickness of 50 μm and without any surface treatment had values of R_a_ and R_z_ of 1.189 μm and 7.623 μm, respectively, characteristic of a relatively smooth and homogeneous surface. After sandblasting, a significant increase in roughness is observed for both parameters, R_a_ and R_z_. With a single sandblasting cycle, the values increase to R_a_ and R_z_ of 6.371 μm and 34.796 μm, respectively, representing an increase of more than five times in R_a_ and approximately four times in R_z_ compared to the untreated surface. This pronounced increase indicates that the abrasive impact of sandblasting generates a much more irregular surface, with high-amplitude peaks and valleys, evidenced by the high R_z_ values and supported by the aforementioned SEM images.

When the treatment was increased to four sandblasting cycles, a slight reduction in roughness values was observed, with R_a_ and R_z_ of 5.745 μm and 31.992 μm, respectively, even though these changes are not statically significant within the standard deviation. In this regard, and comparing with wettability previously discussed, it seems that roughness parameters do not fully reflect the microrelief shapes and redistribution of roughness that affect wettability surface behaviour, highlighting the need for a more detailed roughness profile analysis. This is consistent with the results reported by Kubiak et al. [45], where specimens with similar R_a_ and material-ratio values exhibited noticeable differences in measured contact angles, highlighting that wettability on real engineering surfaces cannot be fully captured by a limited set of average roughness parameters. A similar overall trend was observed for the specimens manufactured with a layer thickness of 100 μm.

#### 3.4.3. Effect of PVD on Roughness Parameters

A PVD coating was applied to femtosecond-laser-modified surfaces, with the aim of evaluating its additional effect on roughness. The values obtained after this treatment are collected in Table 10.

The results indicate that the PVD coating does not substantially change the geometry of femtosecond-laser-textured surfaces but rather acts as a layer that follows the existing surface profile.

For the lowest pitch (0.03 mm), the roughness values after PVD remained low, with R_a_ values between 1.658 and 1.965 μm and R_z_ between 10.870 and 11.855 μm. Compared to femtosecond-laser-textured surfaces without coating, a slight increase in both R_a_ and R_z_ was observed, especially for specimens made with a 50 mm layer thickness. This behaviour can be attributed to the thickness of the coating, which covers peaks and valleys without removing them, slightly increasing the surface profile amplitude.

For intermediate pitches (0.05 and 0.07 mm), the values after PVD coating were similar to, or in some cases slightly lower than, those obtained after femtosecond laser texturing without coating. For example, in specimens with a 50 mm layer thickness and a pitch of 0.05 mm, Ra decreased from 5.520 μm to 4.520 μm, while R_z_ remained at values close to 23–25 μm. This result suggests that PVD can produce a slight smoothing effect on surface irregularities, without substantially modifying the peak-to-valley differences reflected by R_z_.

In the case of the highest pitch (0.10 mm), both R_a_ and R_z_ remained elevated after coating, reaching maximum Ra values of around 5.2–5.7 μm and R_z_ between 25.0 and 27.5 μm. This indicates that the more widely spaced textures generate deep valleys that are not filled or mitigated by the PVD but simply covered by it. As observed after femtosecond laser texturing, the differences between specimens manufactured with 50 and 100 mm layer thicknesses were not significant after PVD coating. There was no clear or systematic trend linking final roughness to the initial layer thickness, confirming that, after femtosecond laser texturing and PVD application, the final topography is mainly controlled by femtosecond laser pitch. This effect contrasts with the behaviour observed on the initial untreated surfaces, where layer thickness was the dominant factor, and shows how subsequent treatments progressively mask the roughness inherent to the manufacturing process.

## 4. Conclusions

This study systematically evaluated the relative influence of VPP processing parameters and post-processing treatments on the wettability of an acrylic resin, establishing a clear hierarchy of effects.

The as-printed surfaces exhibited hydrophilic behaviour in all cases (WCA < 52°). Among the manufacturing parameters, build orientation showed a stronger influence on wettability than layer thickness, with WCA reductions of approximately 43–47% from 0° to 90°. In contrast, layer thickness produced more moderate variations in wettability (typically below 10°), although it significantly affected roughness, increasing R_a_ from 1.19 μm (50 μm layer thickness) to 5.40 μm (100 μm layer thickness). However, this increase in roughness did not directly translate into proportional changes in wettability, indicating that roughness parameters alone are insufficient to explain the wetting response. Post-processing treatments induced substantially greater modifications than printing parameters, becoming the dominant factor governing the final surface behaviour.

Femtosecond laser texturing enabled controlled tuning of wettability over a broad range. At low fluence (F1), the surface became more hydrophilic, with contact angles decreasing to 8–25°, consistent with a Wenzel-like wetting regime. At higher fluence (F2), the behaviour reversed, with WCA values increasing up to 100–108°, corresponding to relative increases of approximately 200%.

The influence of pitch was secondary and did not follow a clear trend, as no direct correlation was observed between pitch (or the associated roughness parameters) and the resulting contact angle. Notably, the highest WCA values were obtained under conditions with relatively low roughness, whereas increasing pitch led to higher roughness without a proportional increase in wettability. This behaviour, together with the strong wettability changes induced by PVD coating despite minimal variations in roughness, indicates that wettability is not governed solely by roughness magnitude, but rather by a combined effect of surface morphology (feature geometry and spatial distribution) and physicochemical surface modifications. The combination of laser texturing and TiN PVD coating resulted in the most significant enhancement in wettability. The coating introduced a strong chemical contribution, increasing WCA values up to 142–154°. Superhydrophobic behaviour (WCA > 150°) was achieved only under specific conditions, namely high laser fluence (F2) combined with pitches ≥ 0.05 mm. Smaller pitches (0.03 mm) resulted in lower WCA values due to partial coalescence of surface features, highlighting the importance of well-defined and spaced microstructures. The PVD coating had only a minor influence on surface roughness, whereas laser-generated topography remained the primary factor governing morphological control. Based on these findings, the optimal conditions for maximising hydrophobicity in this system involve VPP printing at a 45° build orientation, followed by femtosecond laser texturing at a fluence of F2 (0.092 J·cm^−2^) with a pitch ≥ 0.05 mm, and subsequent application of a TiN PVD coating. Under these conditions, WCA values of up to 150–154° were achieved. From a practical standpoint, sandblasting represents a simple and cost-effective method for obtaining moderately hydrophobic surfaces, whereas the combination of laser texturing and PVD coating enables higher-performance and tuneable wettability, suitable for advanced applications, such as self-cleaning surfaces, fluid management, or mould fabrication of hydrophobic parts.

In summary, this work demonstrates that surface engineering strategies can effectively overcome the intrinsic wettability associated with the as-printed state, providing a versatile route to tailor surface behaviour in VPP-manufactured parts. Furthermore, the study identifies the key process parameters required to achieve both hydrophobic and near-superhydrophobic surfaces.

The combined experimental and statistical analysis highlights that the apparent hierarchy of influencing parameters depends on the level of observation and the nature of the evaluated response. From a purely experimental standpoint, layer thickness primarily affects surface roughness but induces only limited direct changes in contact angle. However, when statistical significance is assessed through ANOVA, the relative contribution of this factor is weighted by variability and effect dispersion across the dataset, which explains why certain parameters, such as layer thickness, appear significant despite producing smaller absolute variations.

A similar behaviour is observed for post-processing treatments, where femtosecond laser fluence governs the main wetting transition, while pitch and manufacturing parameters act as secondary modulators depending on the surface state. In this context, statistical significance reflects the consistency and robustness of each effect across all experimental conditions, whereas the experimental trends highlight the magnitude of local changes in wettability. Consequently, both perspectives are complementary: experimental results capture the physical intensity of the wetting modifications, whereas statistical analysis identifies the parameters that most consistently control the overall response. This dual interpretation confirms that wettability is governed by a hierarchical and multi-scale interplay between fabrication conditions, laser-induced surface structuring, and chemical modification induced by PVD coating.

## Figures and Tables

**Table 1:** Mechanical properties of Rigid 10K [21].

Mechanical Properties	Values
Tensile strength	88 MPa
Tensile modulus	11 GPa

**Table 2:** Process parameters of printed samples [21].

	Units	Value	
Printer reference/equipment	-	Formlabs 3BL	
Printing material	VPP resin	10K	
Layer thickness	µm	50	100
Orientation building X plane	º	0, 45, 90	0, 45, 90
External contour exposure (outer perimeter exposure)	mJ/cm^2^	28	47
Infill exposure (internal exposure)	mJ/cm^2^	28	33
Support structure exposure	mJ/cm^2^	56	95
Resin temperature	°C	35	35
Resin recoating speed	mm/s	85	85
Lower roller speed	mm/s	15	15
Waiting time after laser scanning	s	1	1.2

**Table 3:** Water contact angle values of parts manufactured with different layer thicknesses and build orientations. Data are expressed as mean ± standard deviation.

Sample	Water Contact Angles (°)
50_A0	51.7 ± 4.1
50_A45	32.8 ± 3.5
50_A90	27.8 ± 4.9
100_A0	42.8 ± 5.2
100_A45	47.4 ± 2.3
100_A90	24.6 ± 3.5

**Table 4:** ANOVA analysis, fluence vs. pitch.

Factor	F Statistic	Variability (Sum of Squares)	*p*-Value	Significance
Fluence	88.63	36,707.03	2.32 × 10^−11^	Significant
Pitch	0.15	184.31	0.930	Not significant
Layer Thickness	4.30	1779.50	0.045	Significant
Fluence × Pitch	0.81	1003.23	0.498	Not significant

**Table 5:** ANOVA analysis, treatment vs. layer thickness.

Factor	F Statistic	*p*-Value	Significance
Treatment (0/1/4 cycles)	12.78	0.0011	Significant
Layer Thickness	7.40	0.0186	Significant

**Table 6:** ANOVA analysis of all factors.

Factor	F Statistic	*p*-Value	Significance
Fluence	198.4	<0.0001	Highly significant
Pitch	29.5	<0.0001	Highly significant
Layer Thickness	25.0	0.0002	Significant
Fluence × Pitch	15.1	<0.0001	Significant
Fluence × Layer Thickness	10.3	0.0021	Significant
Pitch × Layer Thickness	2.1	0.11	Not significant
Triple interaction	1.5	0.22	Not significant

**Table 7:** Roughness of specimens manufactured with 50 μm and 100 μm layer resolution.

Layer Thickness (μm)	R_a_ (μm)	R_z_ (μm)
50	1.189 ± 0.053	7.623 ± 1.151
100	5.395 ± 0.596	22.252 ± 1.760

**Table 8:** Roughness of specimens manufactured with 50 μm and 100 μm layer thicknesses and different pitches of femtosecond laser modification.

Layer Thickness (μm)	Pitch (mm)	R_a_ (μm)	R_z_ (μm)
50	0.03	1.580 ± 0.089	10.380 ± 0.517
	0.05	5.520 ± 0.335	25.547 ± 1.274
	0.07	5.830 ± 0.655	23.225 ± 1.158
	0.1	6.232 ± 0.165	24.745 ± 1.234
100	0.03	1.591 ± 0.109	9.963 ± 0.497
	0.05	4.957 ± 0.593	25.731 ± 1.283
	0.07	5.544 ± 0.296	24.436 ± 1.218
	0.1	6.325 ± 0.988	27.161 ± 1.354

**Table 9:** Roughness of samples manufactured with layer thicknesses of 50 µm and 100 µm with 1 and 4 sandblasting cycles.

Layer Thickness (μm)	Sandblasting Cycles	R_a_ (μm)	R_z_ (μm)
50	1 cycle	6.371 ± 0.654	34.796 ± 4.814
	4 cycles	5.745 ± 0.332	31.992 ± 2.874
100	1 cycle	5.980 ± 0.580	32.850 ± 3.950
	4 cycles	5.710 ± 0.510	29.600 ± 2.200

**Table 10:** Roughness of specimens manufactured with 50 μm and 100 μm layer thicknesses and different femtosecond laser texturing pitches using a laser fluence F2 (0.092 μJ·cm^−2^), after PVD coating.

Layer Thickness (μm)	Pitch (mm)	R_a_ (μm)	R_z_ (μm)
50	0.03	1.965 ± 0.111	11.855 ± 0.591
	0.05	4.520 ± 0.274	23.828 ± 1.726
	0.07	4.385 ± 0.493	23.480 ± 2.453
	0.1	5.706 ± 0.151	27.508 ± 0.875
100	0.03	1.658 ± 0.114	10.870 ± 0.526
	0.05	5.302 ± 0.634	27.767 ± 2.680
	0.07	5.295 ± 0.283	23.193 ± 1.321
	0.1	5.252 ± 0.820	25.039 ± 0.544

## Data Availability

The data presented in this study are available upon request from the authors.

## Supplementary Materials

The following supporting information can be downloaded at: https://www.mdpi.com/article/10.3390/polym18141738/s1. Figure S1: SEM images of the printing layer thickness (50 and 100 μm) and orientation building of 45° at 100× and 5 kV.

## Abbreviations

The following abbreviations are used in this manuscript:

ABS	Acrylonitrile Butadiene Styrene
AM	Additive Manufacturing
PC	Polycarbonate
PES	Polyethersulfone
PMMA	Poly(methyl methacrylate)
PP	Polypropylene
PVD	Physical Vapour Deposition
SEM	Scanning Electron Microscopy
UV	Ultraviolet
VPP	Vat Photopolymerisation
WCA	Water Contact Angle

## References

[1] Chen B., Dong Z., Jia Y., Li J., Zhang M., Zhang K. (2021). Sepiolite-Based Superamphiphobic Coating with Excellent Robustness, Chemical Stability and Self-Cleaning Performance. Prog. Org. Coat..

[2] Zhang B., Yan J., Xu W., Zhang Y., Duan J., Hou B. (2022). Robust, Scalable and Fluorine-Free Superhydrophobic Anti-Corrosion Coating with Shielding Functions in Marine Submerged and Atmospheric Zones. Mater. Des..

[3] Zhang D., Wu G., Li H., Cui Y., Zhang Y. (2021). Superamphiphobic Surfaces with Robust Self-Cleaning, Abrasion Resistance and Anti-Corrosion. Chem. Eng. J..

[4] Wang J., Wang H., Wang Y., Gao P., Wang F., Men X., Zhang Z., Lu Y. (2021). Design Robust, Degradable and Recyclable Superhydrophobic Materials. Chem. Eng. J..

[5] Chen W., Huang Z., Xiao T., Jiang L., Tan X., Lei Y. (2025). Advancing Superhydrophobic Surfaces: From Mechanism Insights and Design Strategies to Intelligent Applications. APL Mater..

[6] Zaman Khan M., Militky J., Petru M., Tomková B., Ali A., Tören E., Perveen S. (2022). Recent Advances in Superhydrophobic Surfaces for Practical Applications: A Review. Eur. Polym. J..

[7] Cassie A.B.D., Baxter S. (1944). Wettability of Porous Surfaces. Trans. Faraday Soc..

[8] Nomeir B., Lakhouil S., Boukheir S., Ali M.A., Naamane S. (2024). Recent Advances in Polymer-Based Superhydrophobic Coatings: Preparation, Properties, and Applications. J. Coat. Technol. Res..

[9] (2022). Fabricación Aditiva—Principios Generals—Fundamentos y Vocabulario.

[10] Huang S.H., Liu P., Mokasdar A., Hou L. (2013). Additive Manufacturing and Its Societal Impact: A Literature Review. Int. J. Adv. Manuf. Technol..

[11] Bachiller C., Nova V., Ferrer Á., Martínez A., Sandoval N., Marín M.L., Ponce-González L.N. (2023). Additive Manufacturing and Metallization of High-Frequency Communication Devices. Prog. Addit. Manuf..

[12] Lu Y., Chen F., Wu X., Zhou C., Zhao H., Li L., Tang Y. (2019). Precise WEDM of Micro-Textured Mould for Micro-Injection Molding of Hydrophobic Polymer Surface. Mater. Manuf. Process..

[13] Kelly P.J., Arnell R.D. (2000). Magnetron Sputtering: A Review of Recent Developments and Applications. Vacuum.

[14] Pandey K.K., Islam A., Maurya S.S., Raghupathy B.P.C., Sivakumaran M.V., Kavitha N., Keshri A.K. (2023). Hybrid Reinforcement of 1-Dimensional and 2-Dimensional Carbon Nanofillers for Improving the Efficiency of Alumina Membranes. Surf. Interfaces.

[15] Botshekanan N., Majidian H., Farvizi M. (2023). Thin TiN Coating on NiTi Substrate through PVD Method: Improvement of the Wear Resistance. Tribol.-Mater. Surf. Interfaces.

[16] D’Avico L., Beltrami R., Lecis N., Trasatti S.P. (2018). Corrosion Behavior and Surface Properties of PVD Coatings for Mold Technology Applications. Coatings.

[17] Basdeki M., Apostolopoulos C. (2022). The Effect of Shot Blasting Process on Mechanical Properties and Anti-Corrosive Behavior of Steel Reinforcement. Metals.

[18] Drakakaki A., Apostolopoulos C., Katsaounis A., Bjorn H. (2017). Corrosion Resistance and Mechanical Characteristics of Dual-Phase Steel B500c, after Shot Blasting Processes. Int. J. Struct. Integr..

[19] Evans D.C., Lancaster J.K., Scott D. (1979). The Wear of Polymers. Treatise on Materials Science and Technology.

[20] Lin D.-Z.K., Cheng Y.-L., Lin S.-C., Jeng J.-Y.A., Kumar Paral S., Lin D.-Z., Cheng Y.-L., Lin S.-C., Jeng J.-Y. (2023). A Review of Critical Issues in High-Speed Vat Photopolymerization. Polymers.

[21] Formlabs (2022). Ficha Técnica Resina Rigid 10k.

[22] Tumbleston J.R., Shirvanyants D., Ermoshkin N., Janusziewicz R., Johnson A.R., Kelly D., Chen K., Pinschmidt R., Rolland J.P., Ermoshkin A. (2015). Additive Manufacturing. Continuous Liquid Interface Production of 3D Objects. Science.

[23] Jacobs P.F. (1992). Rapid Prototyping & Manufacturing: Fundamentals of Stereolithography.

[24] Melchels F.P.W., Domingos M.A.N., Klein T.J., Malda J., Bartolo P.J., Hutmacher D.W. (2012). Additive Manufacturing of Tissues and Organs. Prog. Polym. Sci..

[25] Bagheri A., Jin J. (2019). Photopolymerization in 3D Printing. ACS Appl. Polym. Mater..

[26] Yuan Y., Lee T.R. (2013). Contact Angle and Wetting Properties. Surf. Sci. Tech..

[27] Nosonovsky M. (2007). Multiscale Roughness and Stability of Superhydrophobic Biomimetic Interfaces. Langmuir.

[28] Lohatepanont M., Chen M., Mendoza Nova L.C., Murray J.T., Merchan-Merchan W. (2024). Exploring Microstructure Patterns: Influence on Hydrophobic Properties of 3D-Printed Surfaces. Micro.

[29] Danielak A., Islam A., Cappelletto N., Agudelo D.M.G., Pedersen D.B. (2024). Design Aspects in Vat Photopolymerization Additive Manufacturing of Hydrophobic Surfaces. 3D Print. Addit. Manuf..

[30] Abo Shawish S.M., Barmouz M., Azarhoushang B. (2025). Feasibility Assessment of Hydrophobic Surface Creation via Digital Light Processing: Influence of Texture Geometry, Composition, and Resin Type. J. Compos. Sci..

[31] Kuisat F., Ränke F., Lasagni F., Lasagni A.F., Kuisat F., Ränke F., Lasagni F., Lasagni A.F. (2021). Simultaneous Micro-Structuring and Surface Smoothing of Additive Manufactured Parts Using DLIP Technique and Its Influence on the Wetting Behaviour. Materials.

[32] Boinovich L.B., Emelyanenko A.M. (2008). Hydrophobic Materials and Coatings: Principles of Design, Properties and Applications. Russ. Chem. Rev..

[33] Sun C., Fang N., Wu D.M., Zhang X. (2005). Projection Micro-Stereolithography Using Digital Micro-Mirror Dynamic Mask. Sens. Actuators A Phys..

[34] Chu W.S., Shehroze M.M., Tran N.G., Dinh T., Hong S.T., Chun D.M. (2024). Green Fabrication of Superhydrophobic Surfaces Using Laser Surface Texturing Without Toxic Chemicals: A Review. Int. J. Precis. Eng. Manuf..

[35] Obilor A.F., Pacella M., Wilson A., Silberschmidt V.V. (2022). Micro-Texturing of Polymer Surfaces Using Lasers: A Review. Int. J. Adv. Manuf. Technol..

[36] Zhi J.H., Zhang L.Z., Yan Y., Zhu J. (2017). Mechanical Durability of Superhydrophobic Surfaces: The Role of Surface Modification Technologies. Appl. Surf. Sci..

[37] Vaida C., Pop G., Tucan P., Gherman B., Pisla D. (2024). Multi-Parametric Optimization of 3D-Printed Components. Polymers.

[38] Li Y., Teng Z. (2024). Effect of Printing Orientation on Mechanical Properties of SLA 3D-Printed Photopolymer. Fatigue Fract. Eng. Mater. Struct..

[39] Gao N., Yan Y. (2009). Modeling Superhydrophobic Contact Angles and Wetting Transition. J. Bionic Eng..

[40] Quéré D. (2008). Wetting and Roughness. Annu. Rev. Mater. Res..

[41] Yong J., Chen F., Yang Q., Hou X. (2015). Femtosecond Laser Controlled Wettability of Solid Surfaces. Soft Matter.

[42] Serles P., Nikumb S., Bordatchev E. (2018). Superhydrophobic and Superhydrophilic Functionalized Surfaces by Picosecond Laser Texturing. J. Laser Appl..

[43] Pazokian H., Selimis A., Barzin J., Jelvani S., Mollabashi M., Fotakis C., Stratakis E. (2012). Tailoring the Wetting Properties of Polymers from Highly Hydrophilic to Superhydrophobic Using UV Laser Pulses. J. Micromech. Microeng..

[44] Oranli E., Ma C., Gungoren N., Heydari Astaraee A., Bagherifard S., Guagliano M. (2024). Sand Blasting for Hydrophobic Surface Generation in Polymers: Experimental and Machine Learning Approaches. Appl. Surf. Sci. Adv..

[45] Kubiak K.J., Wilson M.C.T., Mathia T.G., Carval P. (2011). Wettability versus Roughness of Engineering Surfaces. Wear.

[46] Bechikh A., Klinkova O., Maalej Y., Tawfiq I., Nasri R. (2020). Sandblasting Parameter Variation Effect on Galvanized Steel Surface Chemical Composition, Roughness and Free Energy. Int. J. Adhes. Adhes..

[47] Ensikat H.J., Ditsche-Kuru P., Neinhuis C., Barthlott W. (2011). Superhydrophobicity in Perfection: The Outstanding Properties of the Lotus Leaf. Beilstein J. Nanotechnol..

[48] Pinterich T., Winkler P.M., Vrtala A.E., Wagner P.E. (2011). Experiments on the Contact Angle of N-Propanol on Differently Prepared Silver Substrates at Various Temperatures and Implications for the Properties of Silver Nanoparticles. Atmos. Res..

[49] Bhushan B., Jung Y.C. (2011). Natural and Biomimetic Artificial Surfaces for Superhydrophobicity, Self-Cleaning, Low Adhesion, and Drag Reduction. Prog. Mater. Sci..

[50] Becerra-Borges Y.E., Cazón-Martín A., Etxaniz-Sein U., Manchado J.C., Candal M.V. (2026). Potential Use of Rigid 10K in VPP-UVL Additive-Manufactured Mold Inserts for Injection Molding of Plastic Parts: Opportunities and Challenges. Mater. Des..

[51] Laroche G., Fitremann J., Gherardi N. (2013). FTIR-ATR Spectroscopy in Thin Film Studies: The Importance of Sampling Depth and Deposition Substrate. Appl. Surf. Sci..

[52] Qiao H., Liang G., Shu F., Wang X., Cheng W., Liu J., Wang M., Yang J. (2022). Periodic Surface Nanostructures Induced by Orthogonal Femtosecond Laser Pulses on Tungsten. Optik.

[53] Wu C., Wei X., Chen Y., Liu J., Guo C., Wang Q., Liang S.Y. (2022). Surface Wettability Analysis and Preparation of Hydrophobic Microcylindrical Arrays by μ-SLA 3D Printing. J. Manuf. Process..

[54] Moskal D., Martan J., Honner M. (2023). Scanning Strategies in Laser Surface Texturing: A Review. Micromachines.

[55] Stark B.L., Gamboa M., Esparza A., Cavendar-Word T.J., Bermudez D., Carlon L., Roberson D.A., Joddar B., Natividad-Diaz S. (2024). Materials Characterization of Stereolithography 3D Printed Polymer to Develop a Self-Driven Microfluidic Device for Bioanalytical Applications. ACS Appl. Bio Mater..

[56] Tandogan B., Emir F., Ceylan G. (2026). Effect of Resin Type, Layer Thickness, and Printing Orientation on the Mechanical and Surface Properties of 3D-Printed Occlusal Splints. Polymers.

[57] Vlahou M., Protopapa N., Maragkaki S., Tsibidis G.D., Stratakis E. (2025). Fabrication of Highly Uniform Laser-Induced Periodic Structures on Polycarbonate via UV Femtosecond Pulses. Opt. Laser Technol..

[58] Golhin A.P., Tonello R., Frisvad J.R., Grammatikos S., Strandlie A. (2023). Surface Roughness of As-Printed Polymers: A Comprehensive Review. Int. J. Adv. Manuf. Technol..

