# Supplementary material for: Influence of Additive Manufacturing Parameters and Surface Treatments on Wettability of VPP Acrylic Resins
Source: Polymers (Basel). 2026 Jul 15;18(14):1738. doi: 10.3390/polym18141738 (PMC13418326; doi:10.3390/polym18141738)
Supplement: Supplementary file 1 [file polymers-18-01738-s001.zip › polymers-4324529-supplementary.pdf]

Influence of Additive Manufacturing Parameters and Surface Treatments on Wettability of VPP Acrylic Resins

María Jordá-Reolid[1*], Ivan Dominguez-Candela[2], Mirko Kunowsky[1], Ignacio Sandoval-Pérez[1] and Asunción Martínez-García[1]

[1] AIJU, Technological Center, 03440 Ibi, Alicante, Spain; mariajorda@aiju.es

[2] Technological Institute of Materials (ITM), Universitat Politècnica de València (UPV), Plaza Ferrándiz y Carbonell 1, 03801 Alcoy, Spain

Supplementary Information

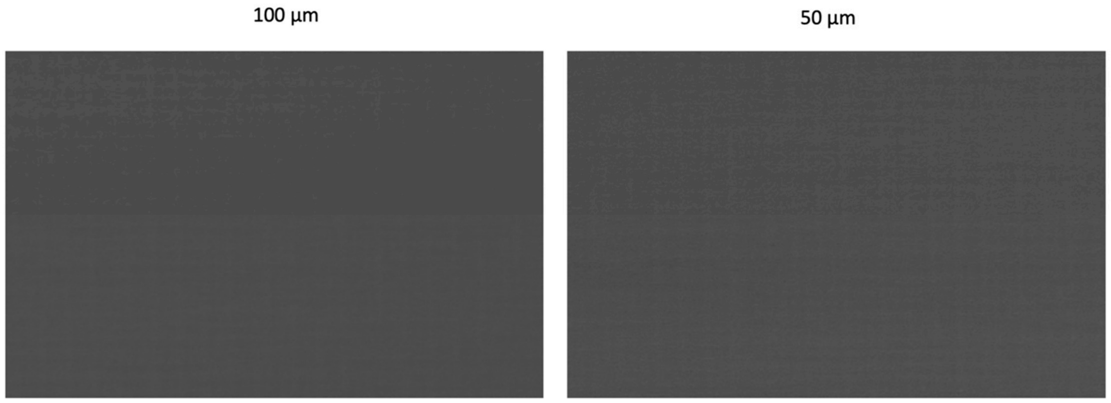

Figure S1. SEM images of the printing layer thickness (50 and 100 µm) and orientation building of 45° at 100x and 5 kV.
